# Experiences of teachers in vocational programmes in special needs schools, City of Cape Town

**DOI:** 10.4102/ajod.v13i0.1333

**Published:** 2024-03-20

**Authors:** Elana T. Solomon, Rosemary Luger, Lieketseng Ned

**Affiliations:** 1 Division of Disability and Rehabilitation Studies, Department of Global Health, Faculty of Medicine and Health Sciences, Stellenbosch University, Cape Town, South Africa

**Keywords:** teacher, challenges, vocational programme, vocational education and training, special needs schools, severe intellectual disability, SID, Differentiated Curriculum Assessment Policy Statement, DCAPS

## Abstract

**Background:**

Vocational programmes run by teachers in the special needs school context can play a significant role in the vocational development of learners with severe intellectual disability (SID). This study aimed to answer the question ‘what are the challenges faced by teachers in the implementation of vocational programmes in selected public special needs schools for learners with SID in the Metropolitan (Metro) District within the City of Cape Town?’

**Objectives:**

The objectives were to describe the challenges as perceived by participants, to highlight common and contrasting challenges in the different schools and to share recommendations on support needed.

**Method:**

A qualitative descriptive study was conducted. A combination of purposive and snowball sampling strategies was used to select six teachers from six special needs schools. One-on-one semi-structured interviews with teachers were performed. An interview schedule was used as a tool and all interviews were transcribed and translated into English verbatim. Thematic analysis was applied.

**Results:**

The findings showed that teachers encounter inadequate resources, a lack of training, and poor support systems. This study highlights the issues of existing policy and the lack of a mandatory policy on vocational programmes in South Africa.

**Conclusion:**

The participants’ experiences added to the existing literature by providing valuable insights into the obstacles teachers encounter in this relatively new curriculum. A multifaceted policy framework that is well funded and implemented is much needed to address the challenges identified.

**Contribution:**

The findings may contribute to the development and strengthening of policies on vocational programmes within the South African context.

## Introduction

The challenges faced by students with disabilities have been drawing attention for quite some time. Unfortunately, individuals with intellectual disability (ID) are underrepresented in the job market worldwide, including in South Africa (Tøssebro & Olsen 2020). In fact, research conducted in Sweden showed that only 22% of people with ID were employed in 2011 (Arvidsson, Widén & Tideman 2015). In comparison, a smaller percentage of people with ID are employed in South Africa than in Sweden (Soeker et al. 2021). Given the critical role that schools play in preparing learners with ID for the workforce and the impact that quality preparation has on post-school outcomes, the government has made vocational training a priority in most schools, including special schools for individuals with disabilities. The United Nations Convention on the Rights of Persons with Disabilities (UNCRPD) advocates for the right of persons with disabilities (PWDs) to access vocational programmes, training, and opportunities to gain experience in the open labour market (United Nations 2006). As a signatory to the UNCRPD, South Africa has committed to implementing these rights within its borders. Additionally, South Africa’s White Paper on the Rights of Persons with Disabilities (Department of Social Development 2015) emphasises the importance of providing equal access to vocational training and reasonable accommodation to ensure that individuals with disabilities can participate in the job market.

Vocational training programmes aim to equip individuals with the skills necessary to secure employment and provide opportunities for people with disabilities to access work (Bartram & Cavanagh 2019; Cocks, Thoresen & Lim Lee 2013). Although research indicates a positive impact of vocational training, the results are not as promising for individuals with intellectual disabilities, who often struggle to find employment after completing these programmes. This is largely because of the poor execution of such programmes. For instance, a study in KwaZulu-Natal, South Africa, found that special schools’ environments pose challenges that hinder learners with special educational needs from being adequately prepared for work (Maniram 2015). Specifically, the study identified a lack of training for teachers to provide the necessary skills, insufficient resources, and inadequate facilities as key issues. The research of Maniram (2015) highlights the significant role that the quality of programme implementation plays in determining the post-transition outcomes of learners.

The first author, an occupational therapist with more than 5 years of experience at a special needs school in the Western Cape province of South Africa, has noticed that implementing a vocational programme can be challenging for both occupational therapists and teachers. The original research included occupational therapists, but this article only reports the findings of teachers. The Differentiated Curriculum Assessment Policy Statement (DCAPS) in South Africa for learners with severe intellectual disability (SID) includes a relatively new vocational programme that has been implemented since 2018. The author, who has interacted with colleagues in various SID schools, has identified common challenges such as limited resources, a lack of support from senior members of the school, learners’ motivation, and finding off-site placements. However, no study has documented this anecdotal evidence. As part of her master’s degree requirements, the author conducted a study to document the challenges and hopes that the findings will assist educational authorities in future planning and policy revisions.

## Literature review

Intellectual disability is characterised by limitations in intellectual functioning and adaptive behaviour, which means that individuals with ID have difficulties in various cognitive abilities and exhibit significant functional deficits (American Psychiatric Association 2013). This disability is lifelong and is one of the most common forms of developmental disabilities, often diagnosed during the developmental period before the age of 18 (American Psychiatric Association 2013). Intellectual disability is typically assessed using standardised psychometric tests.

### Difficulties experienced by learners with severe intellectual disability within the education system

Learners with SID often experience difficulties within the education system. They may struggle with tasks such as reading, written work, and basic mathematics (Adams 2021; American Psychiatric Association 2013). They may also have limited vocabulary and difficulty understanding complex and abstract concepts. Processing information and recalling information from memory are challenging for individuals with SID, and they have reduced ability to comprehend and learn new, complex information. This impacts their performance in the classroom (Cluley 2017). These learners tend to prefer learning through practical applications rather than abstract and complex subjects. They face difficulties in the workplace, particularly with tasks that require extensive problem-solving and steps. Research indicates that individuals with SID often experience poor post-school employment outcomes (Qian et al. 2018). Therefore, it is crucial to provide extraordinary support in various school activities to better prepare them for life. Given the poor employment outcomes, it is essential to consider the education of individuals with SID. Bouck (2014) emphasised the need for critical investigation and research to determine the factors within the school context that contribute to the poor post-school employment outcomes of these learners.

### Legislation and policy on the vocational training of learners with severe intellectual disability in South Africa and South African schooling for learners with severe intellectual disability

Ideologies and policies related to education have an impact on curriculum development across the globe. Historically, individuals with SID were excluded from the education and training systems and experienced social exclusion, discrimination, and marginalisation in both education and employment (Ditchman et al. 2016).

Prior to 1994, South Africa was governed by the Apartheid government, which was notorious for its racial segregation and discrimination against people of colour. This segregation extended to the education sector, where it was based not only on race but also on disability (Engelbrecht 2006).

Following the establishment of democracy in 1994, the new government recognised the need to transform South Africa’s education system and address the discriminatory practices that had excluded individuals with severe disabilities (Rens & Louw 2021).

In post-Apartheid South Africa, inclusive education and training began with the implementation of a single curriculum designed for all learners, including those with ID, with the goal of addressing the inequalities that had arisen from the Apartheid era (McKenzie 2020). However, this curriculum did not adequately address the skills and vocational needs of learners with SID, and therefore was not consistent with the Education White Paper 6: Building an Inclusive Education and Training System. As a result, the South African government introduced a pilot curriculum in 2018 called the draft DCAPS for SID Grade R-5, which is designed for those who have difficulty accessing the academic curriculum.

The DCAPS curriculum is designed to provide home language, mathematics, and life skills education for learners aged 5–18. At the age of 14, additional languages and vocational subjects or electives are introduced, and these subjects are continued until the learner is 18 years old. The vocational subjects covered include agriculture, hairdressing, woodwork, plumbing, arts and crafts, hospitality, office administration, welding, among others. The purpose of this curriculum is to equip learners with practical and vocational skills that will prepare them for employment upon reaching 18 years old. Despite the existence of legislation and wide range of policies in South Africa, the majority of individuals with disabilities, particularly those with intellectual disabilities, remain unemployed in the labour force. Without a robust and well-developed vocational training programme and appropriate support structures, individuals with intellectual disabilities are at a higher risk of unemployment.

#### Role of teachers in vocational programme and the challenges experienced

Vocational programmes are designed to equip individuals with practical skills through the guidance of educators. These practical skills can encompass a wide range of activities, such as cooking, gardening, housekeeping, childcare, welding, motor mechanics, and carpentry. The primary focus of these programmes is to provide knowledge and hands-on training, with theoretical and academic tasks playing a relatively small role (Shaffeei, Razalli & Hanif 2020; Zhang 2009). Educators in these programmes aim to bridge the gap between theoretical knowledge and practical applications in real-world settings.

Several studies conducted across different countries have identified challenges with existing vocational programmes. For instance, research conducted in Minnesota, USA, revealed obstacles such as inadequate student skills, poor family involvement, limited job opportunities, and difficulties in collaborating with businesses (Rooney 2016). Similarly, a study in Saudi Arabia highlighted challenges related to job shadowing and placing learners in the community to gain practical experience as part of the vocational programme. These challenges included limited partnership with employers and a lack of job opportunities for learners in the community. Other obstacles included insufficient administrative support, time constraints between school and job shadowing, safety concerns when taking learners to job shadowing sites, inadequate staff to accompany learners, and limited funding (Almalky 2018). Prior to this study, Kim and Raymond (2010) used the same data collection tool in the USA and ranked barriers from highest to lowest. Barriers were found to include insufficient staff, policy and funding issues. In Saudi Arabia, teachers reported the absence of support from the administration as the most significant obstacle, followed by employers’ poor cooperation with special schools and the lack of cooperation from other community agencies (Almalky 2018). In China, a study exploring the perspectives of Chinese teachers on school-to-work transition services for youth with intellectual disabilities identified learners’ functional limitations, negative community attitudes towards disability, inadequate quality of transition programmes, and inadequate government and societal support as key barriers to successful school-to-work transitions (Xu, Dempsey & Foreman 2014). In Kenya, the educational system has a curriculum to guide teachers in their teaching and learning activities. However, Maina and Muthee (2018) found that many educators lack the necessary training to provide instruction that prepares learners with ID for the future. This deficiency in training negatively affects the delivery of such programmes. Similarly, in South Africa, a study by Maniram (2015) discovered that teachers in Pietermaritzburg, KwaZulu-Natal, faced challenges such as a lack of training, resources, and funds to run programmes for learners with special educational needs. Other obstacles included inadequate parental role modelling, learners’ low socio-economic status, stigma, large class sizes, and negative attitudes from the business community. At the time of the study, there was no formal vocational programme or curriculum for learners with special educational needs, which made it difficult to accommodate their unique learning requirements. It is important to observe that this study was conducted prior to the implementation of DCAPS. According to Vlachos (2008), the lack of vocational training in special needs schools was a significant barrier for learners with ID in South Africa. This issue was identified by teachers of these learners. Ten years later, the introduction of the DCAPS curriculum in South Africa included vocational and skills subjects for SID learners. However, there is limited research on the experiences of those involved in implementing this new curriculum since 2018. Specifically, there are no studies documenting the challenges of implementing this new curriculum of vocational and skills training from the perspectives of teachers in South Africa. The only recent study conducted in South Africa is by Rens and Louw (2021), which focused on teachers’ experiences of implementing life skills in CAPS for learners with SID in special schools. This study found that teachers were facing challenges such as limited resources, the lack of workbooks, heavy workload, and too many assessments. The study suggested that training is necessary for effective implementation of life skills in CAPS.

## Research methods and design

A qualitative descriptive study was conducted to provide in-depth reports of the challenges facing teachers in the implementation of vocational programmes in special needs schools for learners with SID. A qualitative approach involves capturing, exploring and understanding the individual, institution or groups’ experiences and perspectives in depth (O’Leary 2017). This descriptive study was appropriate as it describes the phenomena of interest (Colorafi & Evans 2016), that is, the self-reported challenges experienced by teachers. The objectives were to describe the challenges as perceived by participants, to highlight common and contrasting challenges in the different schools and to share recommendations on support needed.

### Research study setting

The Western Cape province is one of the nine provinces in South Africa and has a population of 7 005 741 million people (Statistics South Africa 2020). The education districts comprise four urban districts and four rural districts (Western Cape Government n.d.). There are 18 public special schools for learners with SID across the Metropolitan (Metro) Districts within the City of Cape Town. The SID schools accommodate learners from 5 to 18 years old. They have specialised and therapeutic support provided by health professionals such as occupational therapists, professional nurses, speech therapists, among others. The number of therapeutic support personnel and number of teaching staff depends on the learner enrolment numbers at each school. The admission criteria include that the learner must have a medical or psychological report stating that the learner has severe intellectual impairment. The school follows the DCAPS Grade R to Grade 5, which is specifically for learners with SID.

### Research study population

The study population for this article were teachers who are involved in implementing vocational training programmes in special needs schools for learners with SID within the Metro Districts of the City of Cape Town. Schools and participants were chosen based on the inclusion and exclusion criteria discussed next.

### Inclusion and exclusion criteria

#### Inclusion criteria

- **Special schools:** Public special needs schools for learners with SID within the metro education district within the City of Cape Town
- Public special needs schools that have a teacher who has provided or is currently providing vocational services.

#### Service providers

- **Teachers:** Teachers who have at least two or more years of experience in providing vocational services in public special needs schools for learners with SID in their current school.
- All genders were included.

### Exclusion criteria

#### Special schools

- Special needs schools for learners with SID within the rural districts in the Western Cape. (I acknowledge that more challenges are encountered in schools in rural areas. However, I wanted to focus on the metro areas, because I work in the Metro District, and I am aware of some of the challenges experienced in the metro, which need to be documented. Given the scope of the small project and because the authors were situated in the metro, they did not have a budget to travel to rural areas).
- Private schools were excluded.

#### Service providers

- Social workers, psychologists or any other professional other than teachers who run vocational programmes and teacher assistants were excluded.

### Recruitment and sampling

#### Special needs schools

All 18 schools were first approached by email. Seven schools expressed interest and 6 out of the 18 special needs schools met the inclusion criteria and participated, including at least one from each of the four districts.

The reason for the small sample size was because of the nature and size of the research project for degree purposes. Furthermore, qualitative studies are not about quantity, but about rich information provided by small number of participants (Bradshaw, Atkinson & Doody 2017; O’Leary 2017).

#### Study participants: Teachers

A total of six teachers volunteered to participate in my study (See Table 1 for a summary and demographic information of participants).

**TABLE 1:** Summary and demographic information of participants – Teachers.

Name of Schools	Pseudonyms	Profession	Type of programme run	Number of years working in vocational programmes in their current school	Gender of participant	Postgraduate studies (i.e. diploma or degree in vocational training and/or rehabilitation)	Courses or workshop or articles or vocational programmes and training from DBE
SID1	Ziqu	Teacher	Hospitality	16	Female	No	CAPS and limited DCAPS training
SID2	Beth	Teacher	Arts and Craft	25	Female	No	Adult education-pottery skills
SID3	Jack	Teacher	Office administration	3	Male	No	No
SID4	Serena	Teacher	Hospitality, Arts and crafts and office administration	16	Female	No	No
SID5	Fiona	Teacher	Nails and beauty, hospitality; Arts and crafts	5	Female	No	DCAPS training
SID6	Ghadija	Teacher	Consumer studies: Needlework	12	Female	No	No training from DBE. Consumer studies at teachers training college

CAPS, curriculum assessment policy statement; DCAPS, differentiated curriculum assessment policy statement; SID, severe intellectual disability; DBE, Department of Basic Education.

### Data collection strategies, data collection tool and process

Upon receiving the signed consent forms, We contacted each participant and asked which method will be convenient for them: video conferencing or face-to-face for me to conduct the interview. Participants were given these two options to choose based on their convenience or in cases of technical issues. Data were gathered via individual semi-structured interviews. The interviews lasted between 45 min and 60 min. Three participants chose video conferencing, and three participants chose face-to-face interviews. All interviews were conducted in English. There was an option if participants preferred the interview to be conducted in a different language in which case a translator would be made available. No participants required a translator. Data vouchers were provided to participants for video conferencing.

During all the interviews, the first author used a predetermined interview schedule that she designed as a tool in order to collect data. Kallio, Pietila and Johnson (2016) said that it is vital to create a semi-structured interview schedule that will provide rigorous data collection to confirm the trustworthiness of the research.

Interviews were recorded using two recording devices as a form of back-up, in case one was not clear or got lost. All recorded data were stored in secured cloud storage with a password for 5 years. Only the first author had the password to access the data.

### Data analysis strategies

We applied the six-step framework as described by Braun and Clarke (2006) to conduct an inductive thematic data analysis. This strategy helped to identify, analyse and see the patterns in the data (Braun & Clarke 2006). Inductive thematic analysis allows data to determine emerging themes. We wanted the data to speak for itself and provide us with the knowledge and themes, rather than trying to fit the data into pre-existing themes. First step was that all interviews were transcribed and translated into English verbatim in order to be familiar with the data, which involves reading and rereading the transcribed data as many times as possible. We also engaged with the data through listening to the recordings and writing notes and ideas for coding. We proceeded to the second step, which is identifying and generating codes using highlighters to code the data. We also created a table to organise and code the data. The third step was extracting the themes, and this was carried out by grouping the coded or relevant data into potential themes. The fourth step was to review the themes, which involved checking if the themes are in line with the coded data and the entire data set. Reviewing and in-depth studying of the themes was performed until data saturation was accomplished and no further themes emerged (Moser & Korstjens 2018). The fifth step was to define and name themes and this was performed by continuous analysis of each theme to give meaning to it and name it. The sixth step was producing a report, which included final analysis, discussion of the analysis in relation to the research question and then producing the report with the findings.

### Ethical considerations

University of Stellenbosch Health Research Ethics Committee granted ethical approval to conduct this study (S21/11/246). Ethical approval was obtained from the Institutional Review Board at a Western Cape public university that approved the research, and ethics consent was received on 03 March 2022. After a letter of approval from the Western Cape Education Department was obtained (reference number: 20220330-1160), the recruitment process commenced from April 2022 to August 2022. The study was conducted according to the ethical guidelines and principles of the international Declaration of Helsinki, the South African Guidelines for Good Clinical Practice (2006), the Medical Research Council (MRC) Ethical Guidelines for Research (2002), and the Department of Health Ethics in Health Research: Principles, Processes and Studies (2015). A written informed consent was provided before the start of the interview and was obtained from all participants. Confidentiality was maintained throughout the study and the identity of participants was kept anonymous. A pseudonym was used as an alternative to the participants’ names on all documents and audio recordings.

### Findings

Through inductive thematic analysis, seven themes emerged from the study data and were aligned with the research aim of the study. The seven themes were then arranged into three overarching themes. The three themes were inadequate resources, a lack of training, and poor support systems.

#### Theme 1: Inadequate resources

This theme speaks about how the lack of resources, facilities and financial resources create challenges for teachers in implementing their vocational programmes at their respective schools.

The participants described their schools as lacking proper facilities, which are equipped like a vocational workshop to enable vocational training. This left them unable to teach or provide training optimally:

‘Um, unfortunately, this is our space [*classroom*]. We’re supposed to have a separate consumer studies room, but it hasn’t come to fruition yet … we don’t have a, a proper consumer study room …’ (Participant 6, Serena, Teacher)

Another teacher expressed a similar problem. She not only teaches hospitality but also nails and beauty, and arts and crafts. Her classroom is not fitted with running water, a washbasin, sink or oven:

‘When I want to do hospitality, I have a very small, you see those small ovens that you can put on the desk, a small oven that, um, I must got fetch in the office every time I need it and sign it out.’ (Participant 10, Fiona, Teacher)

‘I was given a few small basins …, we have to go fetch water outside by the, by the main kitchen and bring it to class.’ (Participant 10, Fiona, Teacher)

The participants experienced challenges in terms of material and financial resources in order to run the vocational programme effectively. It is also evident in the participants’ narrative that they are frustrated by the lack of funding from education management at various levels:

‘We don’t have the resources. It’s a big challenge.’ (Participant 1, Amila, Teacher)

One participant expressed she would buy the materials from her own pocket in order to teach:

‘It’s like, I must go beg for money to buy a kettle, money to buy a set of knives or whatever, you know, whereas that money is due to your elective. You shouldn’t be begging … You know, it’s, it’s really bad … So, I buy my own stuff.’ (Participant 10, Fiona, Teacher)

‘… arts and crafts also, um, very little is given. So, I try to source, or I buy, or I ask for from various places. So, it’s very, very difficult to implement.’ (Participant 10, Fiona, Teacher)

Beth shared that money was given to the school once-off for skills by the Department of Education when the curriculum was first implemented:

‘… the department once gave us the money for the skills and vocational programmes. It was a once off and they never gave us again … it was the first and the last they even given us at all.’ (Participant 2, Beth, Teacher)

Various teachers shared how difficult it is to get funding from the management of the school to buy resources and materials needed for their respective vocational subjects:

‘… the principal, they are refusing to give us money to buy the ingredients or to buy us the material. And it is the problem …’ (Participant 1, Amila, Teacher)

Amila added that it is not about transparency, but rather it is about owning the money that is the problem:

‘I won’t call that transparency because when we doing budget, we’ll do it together. But then when it comes to owning it … Like, like when I’m, when I’m saying owning, uh, I mean like when the money has to be used, there will be no money …’ (Participant 1, Amila, Teacher)

#### Theme 2: A lack of training

Effective implementation of vocational programmes in special schools requires sufficient training for the teachers, to be able to deliver. The participants in this study showed that they have not received adequate training in the vocational programmes and curriculum.

Participants who attended the DCAPS workshop felt that the time allocated for workshops was insufficient:

‘We need a proper training. We didn’t ma get much training … for skills training, there wasn’t much …’ (Participant 2, Beth, Teacher)

‘It was, there was a one-day course when …, it should be a week or a month or some months, but that was a one-day. Everything was just summarised.’ (Participant 2, Beth, Teacher)

The programme also does not come with textbooks. Teachers must do research and find different resources for learners with different abilities to learn and participate, which takes much time and planning:

‘At our school, I don’t have a textbook so that I can have a curriculum in a file. I must obviously get my lesson plans from there, but there’s none, like in mainstream where you have a set textbook.’ (Participant 10, Fiona, Teacher)

#### Theme 3: Poor support systems

Theme 3 focused on inadequate support structures as challenges expressed by participants. The theme was presented and categorised into two sub-themes.

**Sub-theme A: Non-involvement of parents:** Participants shared that non-involvement of parents was a challenge in the implementation. Parents do not attend meetings; parents are not interested and do not ensure carry over at home:

‘The parents’ involvement, our parents are not interested.’ (Participant 1, Ziqu, Teacher)

‘Parents are not fully involved in, in the education of these kids, especially these kids. They don’t care.’ (Participant 2, Beth, Teacher)

Participants described that time for teaching is limited because of issues of transport such as buses being broken down or unavailable:

‘The scholar transport, which the derail is everything when it comes to teaching and learning, teaching, … because you find that the buses are broken, buses are not there. They will be, there are always issues when it comes to the buses. So, learners will arrive late and then they will leave early. So, you cannot finish your programme because of that.’ (Participant 1, Amila, Teacher)

‘… short of the bus buses, the learners have to leave early …’ (Participant 2, Beth, Teacher)

Participants highlighted that they teach in mixed-abilities and multi-grade classrooms – something that they found challenging. This is because there are not enough teachers to allow specialist subjects.

For instance, Gadija has a class with students between 16- and 18 years old for 5 years, where she not only teaches sewing as a vocational subject but also has to incorporate maths, life skills, English and Afrikaans, etc. She expressed the challenge of having to provide different activities to accommodate for all levels of abilities and age groups. This provision of different activities for different abilities has to be in place within each subject she teaches:

‘… intrinsic challenge that’s part of teaching at a school, that the children are all on different levels, you know, mental ability levels and physical ability levels also. I have different levels of ability in how many subjects in English, in Afrikaans, in Maths, in Life Sciences, in Sewing. And I need to cater for all different because you, you know, children are 16, 17, 18 in my class. All levels of functionality understanding. So, I need to do cater for all the levels in Maths and have all different kinds of activities …’ (Participant 12, Gadija, Teacher)

**Sub theme B: Limited support and work or training opportunities in the community:** Work-based learning and/or job shadowing is a part of the vocational programme that gives learners exposure to what it is like in the real working world. However, participants shared experiences of challenges of having to convince the employers to partner with them:

‘… the training part is, is, is the hump you need to get over. You need to convince the manager or manageress of the place that, um, even if you give the child a chance to clean the table, … And then you convince them to, if there’s a spot open, will you allow this child? Takes a lot of work, takes a lot of work.’ (Participant 10, Fiona, Teacher)

The participants added that there is no support or a ‘job coach’ for learners once they are placed in the open labour market or during the transition period:

‘We have to hustle and struggle and, and out of 15, you get two placed maybe for a job, if you’re lucky.’ (Participant 10, Fiona, Teacher)

‘… after school. I mean, when they’re going to the open labour market, it’s not like the workshops who are designed for our children. Open labour market. They just left to their own devices. Ja. And they need constant supervision.’ (Participant 6, Serena, Teacher)

The additional challenge is the lack of accredited certification after completion of the DCAPS programme. The DCAPS curriculum goes up to Grade 5 and is not part of the National Qualifications Framework. Participants expressed challenges in assisting and helping find opportunities for their vocational learners as their learners who are 18-years old and are exiting the school system, leave with a non-accredited school leaving certificate, which makes it more difficult to assist learners in accessing training, learnerships and employment.

Fiona expressed the school leaving certificate:

‘… means nothing. It’s, it’s not even like our grade nine, the one boy he’s also wheelchair bound. Shame. He was in tears … he really wanted to go to [*name of college*] to study further and, or the, the, um, colleges. And he couldn’t because the report says grade five …’ (Participant 10, Fiona, Teacher)

Fiona added that the community has a different mindset and attitudes when they see someone with a grade 5 report, which creates a challenge to give learners opportunities:

‘… how do people out there see that report view that 18 year old. Good grief, you know, an older person that comes from the old standard. I’m gonna look at that report and think, oh, you know, this is standard three mind-set. This child. What can they do?’ (Participant 10, Fiona, Teacher)

## Discussion

A deep analysis was conducted across the themes in this study, revealing the critical issues that teachers face in the implementation of vocational programmes for learners with SID in special needs schools within the City of Cape Town’s Metro District. The study revealed that inadequate resources, a lack of training, and poor support systems are the main challenges that teachers face. To address these challenges, the policies on vocational training programmes for special needs schools in South Africa need to be reformed, and the implementation strategies need to be strengthened.

### Issues of policies and implementation

#### Inadequate resources

The study reveals that one of the key issues preventing teachers from implementing vocational programmes for learners with SID is the scarcity of resources within special needs schools. Participants reported difficulties in obtaining essential resources and funding from their school’s management team (SMT), with some being told that there is no available budget. However, one of the participants, who is part of the school’s management team, expressed that the government only provided a one-time grant for the vocational programme when it was first introduced in 2018, and that the amount was insufficient. Furthermore, the insufficient funding for vocational training programmes from the government is also a common issue in other countries such as the USA, Saudi Arabia, and China (Almalky 2018; Xu et al. 2014). Motala (2006) reported that ordinary public schools in South African townships following an academic curriculum experience inadequate resources, which is consistent with our study of participants in special needs schools in disadvantaged communities. Maaniram (2015) conducted a study on vocational training programmes for learners with special educational needs in KwaZulu-Natal before the implementation of the DCAPS programme. Those schools also faced a lack of financial support from the government, leading to insufficient facilities and resources. This study is consistent with my research on the lack of funding for vocational programmes. It is evident that the shortage of resources is primarily caused by the government’s inadequate funding and mismanagement of financial resources within schools.

Inequalities and uneven resource allocation in special needs schools, stemming from South Africa’s Apartheid history, have contributed to numerous challenges faced by participants in their respective schools. As per Ndimande (2016), special needs schools in black and mixed race communities were underfunded during Apartheid. Despite the passage of time, two schools in townships and three in disadvantaged communities are still deeply affected. Motala (2006) highlighted that underprivileged communities’ ordinary or mainstream schools struggle to secure local funding, a situation also experienced by most special needs schools in this study. Wealthier schools in affluent neighbourhoods, on the other hand, rely on tuition fees, donations and fundraising events, enabling them to acquire necessary facilities and resources, and hire additional staff. This disparity results in under-resourced schools in townships compared to their well-resourced counterparts in more affluent areas because of the economic level of the community around them (Ndimande 2006).

Participants in this study reported that their learners and families come from low socio-economic backgrounds, with high unemployment rates within their special schools’ communities. The lingering effects of Apartheid have perpetuated poverty in these communities, making it challenging for special needs schools to fundraise or obtain local funding. Furthermore, participants cited the lack of proper vocational workshops and facilities as a significant issue, similar to the findings of Maaniram (2015).

According to Maaniram’s (2015) research, two out of three special needs schools for learners with special educational needs in Pietermaritzburg have adequate infrastructure, which allows for optimal teaching and learning. As a result, learners with different disabilities have a better chance of acquiring skills. No information was provided on the source of funding for the successful implementation. However, one of the schools in the study lacked specialist rooms and facilities, which limited teaching and prevented learners from developing their skills. It is unclear whether this school was located in an urban, rural, or township area of Pietermaritzburg. My research study contributes to the literature by highlighting the challenges faced by teachers in special needs schools for learners with SID in implementing the DCAPS programme. Furthermore, my findings provide a comprehensive description of the challenges faced by special needs schools for SID in Cape Town, including two from townships and three in disadvantaged communities from the post-Apartheid era. Despite government policies aimed at addressing resource inequalities in South African schools, there is a lack of funding for special needs schools in townships and disadvantaged communities, as well as for those with diverse socio-economic backgrounds. In addition, policies on funding for vocational programmes, specifically in special needs schools, are lacking. Vocational programmes require substantial funding for materials, equipment, maintenance, and resources to ensure effective implementation. Special schools cater to learners who have high support requirements, which can result in additional expenses for the school, such as acquiring assistive devices and hiring assistants to aid in the teaching and learning process for learners with SID.

### A lack of training

The shortage of training for teachers in special needs schools who teach vocational programmes in South Africa is because of unclear policies. When the DCAPS was introduced as a pilot project, there were no comprehensive training guidelines provided for teachers. Among the six teachers in this study, five reported that the training they received was insufficient. This finding is consistent with Cotton (2000), who emphasised the lack of training for vocational teachers working with children with special needs, which ultimately leads to many of the challenges teachers face in implementation. As a result of inadequate training, the current participants have a challenging time differentiating the DCAPS curriculum, integrating subjects, and creating various resources to accommodate different levels and needs. Participants feel overwhelmed by working with learners who have SID and exhibit diverse levels of functioning, behavioural, and emotional issues, which are different from learners without ID. The lack of understanding of learners with SID and difficulty in planning and managing them were apparent in their narratives.

The issue of inadequate training is prevalent in studies that highlight poor teaching methodologies and difficulty in differentiating the curriculum for learners with diverse needs. This problem seems to be common among teachers in other special needs schools in both developed and developing countries (Ahammed 2021; Horne & Timmons 2009). Research conducted in Swaziland, a low-income nation, found that teachers in inclusive educational environments face similar challenges when working with children with various needs and multiple disabilities, feeling unsupported and inadequately trained to provide the necessary support to learners (Bagree & Lewis 2013).

The challenge of differentiating instruction for learners with SID is further exacerbated by a lack of understanding of SID among some participants in the study. It seems that some teachers adopt a mainstream approach when teaching learners with SID, which may be attributed to the insufficient support and inadequate subject advisors in special needs schools. These advisors are responsible for offering curriculum guidance and specialised training in the specific subjects that teachers teach.

### Poor support systems

The reasons for the absence of support systems are multifaceted and intricate. This issue can be further categorised into four areas: the school level, community level, institutional and political level. Firstly, the absence of support systems is because of unclear policies in the drafted DCAPS curriculum (vocational programme) regarding roles, responsibilities, and support needed to achieve its objectives. Secondly, there are no rules or plans outlined in educational policy documents and guidelines for monitoring and ensuring the availability of support for the implementation of the curriculum and/or vocational programme.

#### School level

Regarding the school level, research by Rooney (2016) emphasises the significant role that parental involvement plays in reinforcing learning, providing learners with opportunities to grow in their environment, and facilitating the transition from school to work. In this study, when parents were not involved, implementing vocational programmes for participants became challenging. The participants reported that parents neither attend meetings nor complete practical assignments at home, making it difficult to effectively implement the programme. These findings align with those of Rooney (2016). Furthermore, Maniram (2015) in Pietermaritzburg, KwaZulu-Natal, South Africa, noticed that the lack of parental involvement in learners’ environments hinders preparation for employment. It is worth noting that various studies worldwide note that learners face different contextual challenges that affect the lack of family involvement (Humphrey-Taylor 2015). Research indicates that the reasons for insufficient parental participation from disadvantaged communities are rooted in low socio-economic context, limited vision, work obligations, family dynamics, educational level, and a lack of empowerment (Munje & Mncube 2018). These factors may explain why study participants are experiencing a lack of parental involvement, as many of their learners and parents come from disadvantaged communities, contributing to poor implementation. The ongoing systemic issues such as the legacy of Apartheid continue to perpetuate poverty and exacerbate existing problems.

One of the challenges faced in the implementation of vocational programmes is the shortage of specialised workbooks and textbooks. Participants in the study reported that the new DCAPS programme did not provide textbooks, making it difficult for them to access resources for different levels of functioning. According to the participants, using workbooks is crucial as without it, extensive research, planning and time is required. This finding aligns with the research conducted by Rens and Louw (2021) on teachers’ experiences in implementing the Life Skills CAPS curriculum for learners with SID in both well-resourced and under-resourced schools in the North West province of South Africa. They concluded that the lack of specialised books, particularly for the SID curriculum, hinders implementation and poses a significant problem for teachers. Although not specific to vocational training, this study highlights the link between the absence of workbooks and poor implementation. It remains unclear why the Department of Education in South Africa did not provide textbooks for the DCAPS programme, but this may be because of poor planning, as the policy document was not well-structured for learners with SID. For instance, in the hospitality programme, the teachers were instructed to start cooking or baking with the learners in week two, without any prior introduction to basic hygiene or familiarisation with the equipment and materials required for the task.

According to the research, there is a problem with the school not following a budget or misusing funds, which leads to the participants being unable to receive the money they were expecting to purchase materials. The findings also showed that a culture of honesty, transparency, and openness is lacking in the school. Although there were limited studies available in the literature on this topic related to special schools, Metsy (2006) and Rangongo et al. (2016) found that school principals and governing bodies rarely consult with teachers on financial matters related to the school, which affects the school’s ability to achieve its educational objectives. Aina and Bipath (2020) also stated that a lack of openness and involvement of others in financial management mostly occurs in schools situated in townships. Another possible explanation for the misuse of funds could be the school’s tight budget, especially in disadvantaged communities, which may require prioritising or redirecting funds where they are most needed.

#### Community level

This study identified a difficulty in persuading employers within the community to accept learners with SID for job shadowing, which presents a challenge for participants in accessing job shadowing and work opportunities and, consequently, makes the implementation of the programme challenging. Participants in this study indicated that employers are reluctant to accept learners from special needs schools for job shadowing. These findings align with those reported by Almalky (2018) in Saudi Arabia, who found that teachers reported that the community was not supportive, and employers were not willing to collaborate with schools. This was identified as a significant barrier to the implementation of community-based vocational education (job shadowing).

The lack of policy in South Africa that provides job shadowing training for learners with SID contributes to the difficulty in convincing employers and dealing with the community, who are not supportive in accepting learners for job shadowing. The absence of policy outcomes, advantages of job shadowing programmes, well-defined roles for special needs schools and employers, incentives, and support (such as supervisor and protection for the employer) exacerbates the issue.

Additionally, studies have shown that the scarcity of work opportunities makes it challenging for participants to access job shadowing and work placements and, consequently, presents a challenge in implementing the programme. According to Rooney (2016) in the USA and Almalky (2018) in Saudi Arabia, limited work opportunities pose a significant obstacle, while Xu et al. (2014) from China also experienced that the lack of work opportunities served as a barrier in the implementation of their vocational programmes. The problem of limited employment prospects in this study is largely influenced by the historical legacy of Apartheid, which forced people of mixed race to be segregated and reside in townships and disadvantaged areas located far away from the city, where there are very few opportunities available. In South Africa, there is a shortage of jobs, and both the workforce and businesses are not sufficiently aware of the needs of individuals with disabilities, such as ID.

#### Policy level

Curriculum policies and guidelines play a critical role in providing direction to those responsible for implementing programmes. However, the existing policies on vocational training programmes for special needs schools in South Africa catering to learners with SID are insufficient. This results in unclear roles, responsibilities, accountabilities, and processes. In this study, participants highlighted that a lack of adequate staff poses challenges in implementation. The presence of a job coach or supervisor as part of the process was considered vital as school-leavers with SID require additional support during the transition period. These findings align with Almalky (2018), who reported insufficient staff as a barrier to implementation. Earlier, Xu et al. (2014) identified inadequate support as a challenge and emphasised the need for specialised job officers to assist learners with SID in adapting to the open labour market.

This research highlights the substantial difficulties that participants experience in helping and supporting the job placement process. According to Aston, Banks, and Shevlin’s (2021) study, individuals with intellectual disabilities face significant obstacles in accessing post-school opportunities because of the lack of formal, recognised qualifications or certifications. Vlachos’ (2008) research in South Africa revealed that the majority of individuals with intellectual disabilities cannot enrol in the Skills Education Training Authority (SETA) programmes because of the absence of Grade 9 qualifications. There is a scarcity of studies in other parts of the world that examine the challenges teachers face in facilitating placement for learners who lack an accredited school leaving certificate. This issue stems from South Africa’s policy not clearly addressing the inclusion of individuals with SID in the National Qualifications framework, and a lack of policy to strengthen the implementation of this process, allowing individuals with SID to acquire an adapted accredited certificate that accommodates their skills and abilities.

### Limitations

A drawback of this study is the scarcity of prior research on the subject within South Africa. Since the implementation of the DCAPS in special needs schools for learners with SID began in 2018, there is a lack of available and published literature on the topic. As a result of constraints such as budget, scope, and timeframe, it was not feasible to interview teachers representing each of the 16 electives and/or vocational subjects. Furthermore, schools in rural areas were not included in the study, which may have provided valuable insights into the challenges they face.

### Recommendations

- Taking into account the cost of operating a vocational programme within special needs schools that require high levels of support, the South African Government should allocate extra funding to the Department of Education to provide the Provincial Departments with the necessary resources and facilities for learners with SID.
- A needs analysis should be conducted by trained personnel from the Department of Education at special needs schools, and a financial budget should be compiled and submitted to the South African Government. The aim is to raise awareness of the practical costs and expenses involved in running vocational programmes for learners with SID in special needs schools. It is suggested that the government revise the budget in light of these findings.
- In order to effectively address the unique needs of the South African context, a comprehensive, multifaceted policy framework should be implemented and pilot-tested using evidence-based principles. This framework should encompass not only formal curriculum instruction, such as DCAPS, but also a range of support systems that are essential components of a vocational programme, including transition support personnel (job coaches).
- The government could require that all special needs schools with SID implement vocational programmes under a policy framework that includes comprehensive guidelines and support. This framework should outline the roles and responsibilities of the vocational and placement committees within the schools, as well as the accountabilities, funding allocation, and transparency processes that are necessary for success. The framework should be mandatory by government and include all necessary documentation to ensure that the vocational programmes are implemented effectively.
- Legislation of a curriculum for learners with SID and implementation of an accredited or recognised certificate upon completion of a vocational programme should be added to the existing policies and guidelines.
- The government should allocate financial resources to support a comprehensive national or provincial vocational and transition assistance programme, which will enhance intersectoral cooperation among educational institutions, agencies, local companies, and the broader community. To encourage participation and motivation from companies, businesses, and the community, additional incentives should be provided for hiring individuals with SID when they partner with these programmes.
- It is recommended that research study similar to this study be conducted nationally in the future, as this study only focuses on the Metro Districts within the City of Cape Town of the Western province. Furthermore, it is suggested that rural schools be included in the research, as they can offer valuable insights into the unique challenges they face. Investigating the cost of running vocational programmes, such as hospitality and agriculture, and comparing them to the current funding allocated to schools can provide valuable data regarding funding allocation.

## Conclusion

The study identified several challenges faced by teachers in implementing vocational programmes for learners with SID in the Metro District of Cape Town, South Africa. Specifically, inadequate resources, a lack of training, and poor support structures were identified as key issues. In addition, the study highlighted the need for stronger policies and clearer guidelines on vocational programmes in special needs schools. It is recommended that the South African government develop and strengthen such policies to improve the implementation and provide increased support structures for teachers and learners. A multi-faceted policy framework that is tailored to the South African context should be implemented and piloted based on evidence-based principles.
